# Abstracts DGG

**DOI:** 10.1515/iss-2019-2003

**Published:** 2019-03-20

## DGAV: Gender medicine

### Gender Gap in European Surgical Literature in the 21st Century

(Abstract ID: 840)

I. Kerschbaumer^1^, A. Dubecz^1^, N. Solymosi^1^, H. J. Stein^1^

^1^*Klinikum Nürnberg Nord*

**Background:**

After decades of underrepresentation, women today globally outnumber men in medical universities. In 2017, Germany reported that 61.1 percent of medical students were female. Similar trends were seen in the United Kingdom and the United States, which reported female students represented 59.0 percent and 50.7 percent of their student populations respectively. However, despite this steady growth of female student rates, some medical fields remain solely male dominated. While in 2017, 47.7 percent of women made up almost half of all practicing doctors in Germany, only 20.4 percent of all German surgeons were female. Furthermore, a significant disparity is seen when comparing the ratio of men to women in medical executive positions. As publication and professional progression are unequivocally linked, we retrospectively analyzed the quantity and ratio of women in professional European literature.

**Materials and methods:**

Two leading surgical Journals ("Der Chirurg" and "British Journal of Surgery") were selected by their impact factor and articles analyzed by gender of publishing authors in the years 2005, 2010, 2015 and 2017. For comparison the Journal of Internal Medicine ("Der Internist") was also evaluated. The articles were categorized, and gender of authors determined by Internet search. Moreover, in case of bi-sexual names we relied on institutional websites and their respective employee indices. Percentages of female authors were then compared to proportions of women working in the respective fields, by analyzing public records.

**Results:**

Gender was determined in 96.6 percent of all cases. While only 18 percent of all analyzed authors were female, upward trends in the number of female authors were determined in all European journals. In the journal of internal medicine, "Der Internist", the number of female authors increased significantly from 6.9 percent in 2005, to 23.7 percent in 2017. The German based surgical magazine, "Der Chirurg", increased the number of females it published from 11.1 percent in 2005 to 14 percent in 2017. The "British Journal of Surgery" shows an increase in female authors from 12.7 percent in 2005, to 22.3 percent in 2017. In all analyzed years women were most underrepresented in "Der Chirurg", with 13.3 percent of all authors being women, followed by "Der Internist" (18.9 percent) and the "British Journal of Surgery" (23.6 percent).

**Conclusion:**

Across the board, the number of females in medicine is increasing. While more females in medicine means more opportunities for publications, women do not participate in research as frequently as their male peers. Surgical disciplines continue to have few women contributing to scientific research. Further studies will be necessary to elucidate the causes of this gender gap and create improved opportunities for women to succeed in a surgical, as well as research environment.

## DGAV/DGG/DGOOC/DGU: Allrounder or specialist: Vascular Surgery Training for Visceral/General Surgeons

### Leiomyosarcoma of the inferior vena cava - a rare tumor entity

(Abstract ID: 239)

K. Keller^1^, J. De Deken^1^, D. Schubert^2^, T. Petzold^1^, G. A. Stavrou^1^

^1^*Klinikum Saarbrücken*

^2^*Krankenhaus St. Elisabeth & St. Barbara, Halle (Saale)*

**Background:**

Leiomyosarcomas are rare neoplasias wich are generally localized in the gastrointestinal tract and the retroperioneum. Among the rare primary vascular tumors, leiomyosarcomas are one of the most frequent malignant entities. Due to the slow growth, the tumors remain clinically inapparent for a long time and are usually only diagnosed in a locally advanced tumor stage.

**Materials and methods:**

Due to theheterogeneous presentation of these tumors, a multitude of different surgical resection an reconstructive procedures have been suggestet as treatment options. Our patient had an extensive intramural and intraluminal tumor manifestation and underwent a segmental resection of the vena cava. Reconstruction was achieved by implanting a PTFE prosthesis. Postoperatively an asymptomatic stenosis developed due to a pericaval hematoma with consecutive compression of the prosthesis. An angiographic implantation of a stent was successfully performed.

**Results:**

The patient had no symptoms or tumor recurrence in the 24-month follow-up<

**Conclusion:**

The clinical symptoms of leiomyosarcoma of the vena cava are often unspecific. Despite modern imaging techniques, the diagnosis can often only be made peroperativly. The therapy of choice consists of radical en bloc tumor resection wich enables a 5-year survival rate of almost 50% for R0 resections. Due to a variety of topographic and tumor biological manifestations, no standard has been established for the resection of leiomyosarcomas. Taking these criteria into account, the extent of the resection should be planned individually.

## DGG: Organizational structures in vascular medicine: vascular center, specialized center, MVZ

### Impact of weekend treatment on short-term and long-term survival after urgent repair of ruptured aortic aneurysms in Germany

(Abstract ID: 826)

C.-A. Behrendt^1^

^1^*University Medical Center Hamburg-Eppendorf (UKE)*

**Background:**

There is some evidence that weekend admission to the hospital is associated with worse outcomes compared with weekday admission. However, only a few studies have focused on weekend vs weekday surgery outcomes. This study aimed to determine whether there is a weekend effect on outcomes in the treatment of ruptured aortic aneurysms in Germany.

**Materials and methods:**

Health insurance claims of Germany's third largest insurance provider, DAK-Gesundheit, were used to investigate short-term and long-term mortality after weekend vs weekday treatment of ruptured aortic aneurysm. Patients undergoing endovascular repair (ER) or open surgical repair (OSR) between January 2008 and December 2016 were included in the study. Both propensity score matching and regression methods were used to adjust for confounding.

**Results:**

There were 1477 patients in the cohort, of whom 517 (35.0%) underwent ER and 960 (65.0%) OSR. Overall, 995 (67.4%) patients underwent an operation on weekdays (Monday to Thursday), and 482 (32.6%) patients underwent an operation on a weekend (Friday to Sunday). In multivariable models, patients who underwent an operation on a weekend were at higher risk of in-hospital death after OSR (49.2% vs 38.0%; odds ratio [OR], 1.61; P = .001), and there was a trend toward higher in-hospital mortality after ER (29.5% vs 21.2%; OR, 1.55; P = .056). The ER of thoracic or thoracoabdominal aortic ruptures was associated with significantly higher in-hospital mortality compared with ER of abdominal aortic aneurysm (OR, 1.69; P = .026).

**Conclusion:**

Weekend repairs of ruptured aortic aneurysms are associated with worse in-hospital survival compared with weekday surgery. ER of thoracic or thoracoabdominal aortic ruptures is associated with worse in-hospital survival compared with ER of ruptured abdominal aortic ruptures. This might be an international phenomenon requiring joint learning and action in times of centralization of aortic procedures.

## DGG: Endovascular and open treatment

### Questionable interventional stent angioplasty; long segment stenting of the superficial femoral artery and popliteal artery

(Abstract ID: 23)

A. Dadras^1^, C. Uhl^1^, T. Betz^1^, I. Töpel^1^, M. Steinbauer^1^

^1^*KH Barmherzige Brüder, Regensburg*

**Background:**

Stenting of the femoropopliteal segment is a common procedure for vascular occlusion and stenosis in peripheral artery disease. Initially, long segment stenting was described in percutaneous coronary interventions. Eventually, the method was adopted for the endovascular treatment of the femoropopliteal lesions.

**Materials and methods:**

We present two cases, one presenting with severe ischemia and the other with stent infection after a long segment stent angioplasty of the superficial femoral artery and the popliteal artery. Hospital-stay documents and data were collected from two patients. A systematic search of the Medline database was performed with the purpose of identifying other similar cases and results.

**Results:**

Bypass surgery was performed in both patients. Additionally a complete surgical removal of the stents was performed in the patient presenting with infection. Autologous vein was the used graft material in both cases. Follow up showed patent bypasses without signs of reinfection or wound complications. Due to the severe ischemia in the one case a minor amputation was necessary.

**Conclusion:**

Advances in the endovascular techniques offer new possibilities in the treatment of long segment femoropopliteal lesions. However, severe complications could also occur after endovascular therapy.

### Limb occlusion following endovascular aortic repair: Prevention and treatment

(Abstract ID: 446)

K. Meisenbacher^1^, M. Wortmann^1^, P. Contrin^1^, D. Böckler^1^, P. Geisbüsch^1^

^1^*Universitätsklinikum Heidelberg*

**Background:**

Limb occlusion is reported to be up to 7% following endovascular aortic repair (EVAR). It is thereby one of the most common complications requiring secondary interventions. Patients with limb occlusion present in 50% of the cases with critical limb ischemia and have a high mortality rate of up to 15%. Prevention of limb occlusion and its successful treatment is thus of key interest. The aim of this review abstract was therefore to describe risk factors for limb occlusion, highlight strategies for prevention and develop a treatment algorithm involving current open and endovascular modalities.

**Materials and methods:**

A selective literature research on risk factors and treatment modalities of limb occlusion after EVAR in the light of current guidelines and expert recommendations was performed.

**Results:**

Selective literature research showed five risk factors for limb occlusion after EVAR: (1) iliac angulation > 60°/high iliac tortuosity (OR: 5.76), (2) Severe iliac calcification (OR 5.8), (3) Excessive oversizing > 15% (OR 5.54), (4) limb placement in external iliac artery with (5) external iliac artery diameter < 10 mm. Limb occlusion could be attributed to a technical error during planning and implantation in 60% of the cases. Especially in patients with the aforementioned risk factors, limb occlusion rates can be minimized by accurate and detailed planning of the EVAR-procedure, profound knowledge of stentgraft- and limb designs, use of endovascular adjuncts and intraoperative 3-D imaging for early detection of limb complications before the occlusion occurs. While there are multiple treatment modalities (open, endovascular & hybrid procedures) for limb occlusion after EVAR, recommendations concerning therapy selection are diverse. Even in the endovascular era, open surgical repair with iliaco-femoral/femoro-femoral cross-over bypass is used in 60% of the patients. Re-occlusion after initial successful treatment with endovascular and/or hybrid repair still occurs in up to 30%. Our algorithm favors an endovascular treatment in patients with intraoperative limb occlusion and patients with an early onset of symptoms and mild ischemia.

**Conclusion:**

Patients with tortuous iliac arteries, significant iliac vessel calcification that require limb placement in small diameter external iliac artery are at highest risk for limb occlusion. During planning and implantation, a special focus on prevention strategies of this complication should be made. While there are multiple treatment modalities for limb occlusion after EVAR open bypass surgery remains a fast, safe and durable solution used in most patients even nowadays.

### Extensive endovascular repair of aortic disease: from the aortic arch to the iliac arteries

(Abstract ID: 611)

N. Tsilimparis^1^, K. Spanos^2^, S. Haulon^3^, N. Dias^4^, F. Rohlffs^2^, T. Kölbel^2^

^1^*Uniklinik München, München*

^2^*Deutsches Aortenzentrum Hamburg*

^3^*Hôpital Marie Lannelongue, INSERM UMR_S 999 - Université Paris Sud*

^4^*Skåne University Hospital Malmö*

**Background:**

Extensive endovascular repair (aortic arch to iliac arteries) of the aortic disease has not yet been fully evaluated, while the experience derives from few centers. The aim of our study was to evaluate patients that underwent extensive endovascular aortic stent graft coverage in terms of early- and mid-term clinical outcomes.

**Materials and methods:**

A retrospective multicenter study of prospectively collected data was undertaken. All patients were treated with extensive endovascular aortic stent graft coverage in one or two stage procedure, using the Cook Medical (Bloomington, IN, USA) fenestrated and/or branched endografts at three experienced endovascular centers. Demographic data, past medical history, intra- and peri-operative details, and early post-operative morbidity and mortality were also recorded. Patients were followed up clinically and with computed tomography angiography (CTA) which wasscheduled postoperatively, at 6 and 12 months, and annually thereafter.

**Results:**

Between 2012 and 2017, 33 patients (22/33 males) were treated with fenestrated/branched EVAR (fbEVAR) and fenestrated/branched TEVAR (fbTEVAR) resulting in extensive aortic stent graft coverage. Most of the patients (20/33) had as a second stage procedure the endovascular repair of the thoraco-abdominal aortic disease, while 3 patients had a one-stage procedure. The mean patients’ age was 66.5±12.6 years, and the mean interval between the procedures was 13±12 months (last the arch-thoracic intervention; 21±15 months vs. the thoraco-abdominal intervention; 9±9.5months, p=0.021). Demographics and co-morbidities were similar in all groups. Most of the patients were asymptomatic, and one-third of the patients had an aortic dissection. For the arch-thoracic intervention 20 fenestrated and 13 branched devices were used, while for the thoraco-abdominal intervention 23 fenestrated, 5 branched ones and 5 composite devices. The use of spinal drainage was more common in thoraco-abdominal interventions (20/33). In 18 patients an LSA revascularization was undertaken before the arch intervention. The technical success was 100%. There was no intra-operative death. Intra-operative details were similar between two-stage groups in terms of duration, contrast volume, radiation dose and fluoroscopy time. The mean hospital stay was 15.2±13.5 for the arch-thoracic intervention and 12.3±9.2 for the thoraco-abdominal intervention. Two patients died in hospital after the thoraco-abdominal intervention, presenting 6% (2/33) of total 30-day mortality. Four patients developed SCI (12%; 1 permanent), while one patient developed MI. At first month’s CTA, 4 patients developed type Ia Endoleak, 1 type III and 5 type II. The mean follow up was 23±16.7 months; 6 patients died. The survival at 12 months after the second procedure was 72% and the freedom from any re-intervention was 82%. Freedom from arch-thoracic re-intervention was 87% at 12 months. Freedom from thoraco-abdominal re-intervention was 81% at 12 months.

**Conclusion:**

Extensive endovascular coverage of the aorta for aortic pathologies seems to be a safe procedure, with low peri-operative morbidity and mortality, demonstrating good early and mid-terms outcomes in terms of mortality and re-intervention.

**Picture:**
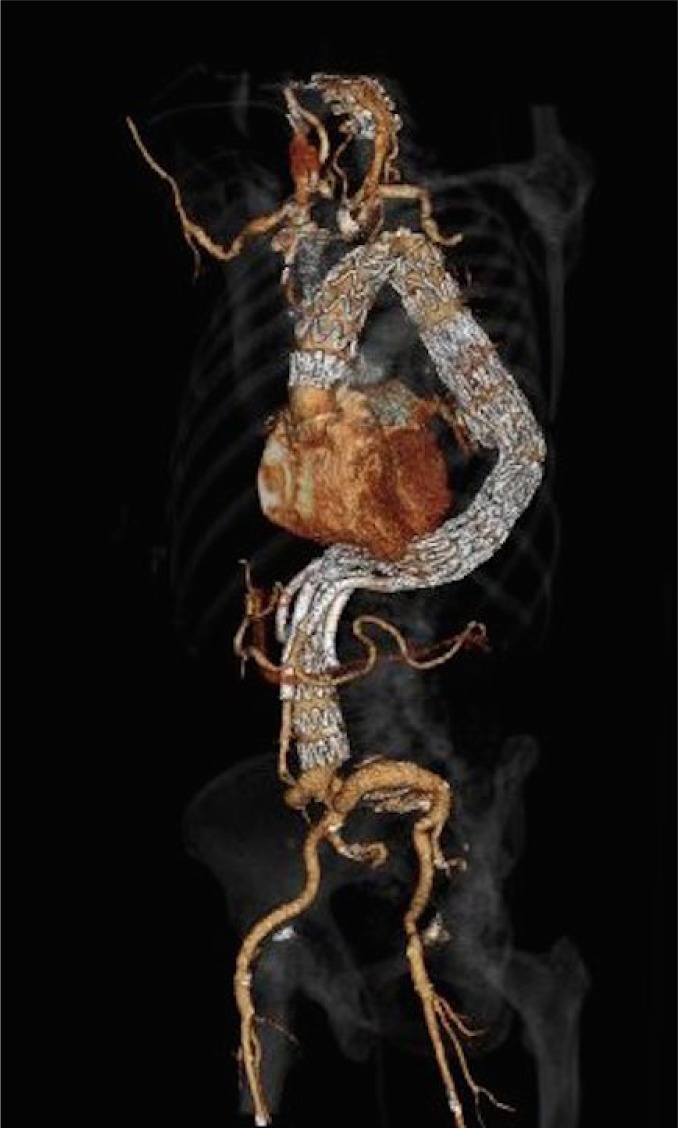


### When EVAR is not an option, open repair of a juxtarenal aortic aneurysm with an underlying horse-shoe kidney becomes the only resort

(Abstract ID: 835)

A. Al Halabi^1^, U. Brune^1^

^1^*Allgemeines Krankenhaus Celle*

**Background:**

Horseshoe kidney is the most common congenital kidney anomaly. It is defined as a medial fusion of the kidneys, mostly anteriorly to the aorta. Its coexistence with an abdominal aortic aneurysm is extremely rare. It presents however a serious challenge in endovascular as well as open repair.

**Materials and methods:**

We present a 72 year old male who was referred to our clinic for the assessment of an incidentally diagnosed asymptomatic aortic aneurysm with an accompanying horse-shoe kidney. A CT- Angiography demonstrated a maximum diameter of 60 mm with juxtarenal location, considerable kinking and bilateral aberrant renal arteries. The left and right common iliac arteries were also aneurysmatic, with diameters of 2.6 and 2.3 cm respectively.

The previous medical history of the patient included arterial hypertension, smoking and an open cholecystectomy due to a perforated cholecystitis ten years ago. The patient was in a good general condition. Except for a diastasis recti, there were no relevant clinical findings.

Considering the unsatisfactory landing zone and configuration of renal arteries, none of the EVAR solutions available then, including fenestrated and branched stent-grafts, were suitable. The patient was thoroughly assessed and considered fit for open repair, and he was consented.

The operative approach was initiated by a median laparotomy and a transperitoneal approach. After consulting our urologists, a midline sectioning of the Isthmus of the horse shoe kidney was carried out using monopolar cautery and a continuous PDS sutures of both stumps, followed by the exposure of the aorta from the renal arteries proximally to the common iliac arteries distally.

The aneurysmatic Aorta and iliac arteries were replaced with an 8mm Dacron bifurcated graft. A left accessory renal artery was reimplanted onto the main body of the graft, while the right renal artery was transpositioned onto its right branch over a Dacron interponate.

**Results:**

Postoperatively the patient recovered well and was discharged with good renal function 14 days later.

One-year follow-up revealed no complications other than an incisional hernia, which was laparoscopically treated.

**Conclusion:**

The superiority of EVAR to open repair is in most cases inarguable. This case however emphasizes the irreplaceable role of open aneurysm repair in certain cases where the EVAR is not feasible and illustrates the importance of a multidisciplinary approach to achieve the best treatment outcomes.

**Picture:**
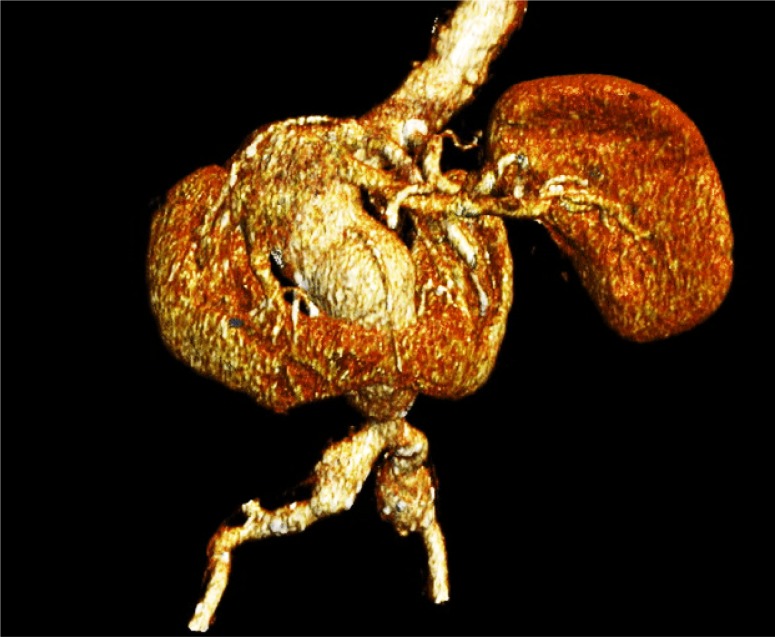
Figure 1: A computer-assisted 3D Reconstruction of the CT-Findings illustration the Aneurysm and horse-shoe Kidney

## DGG: Emergencies and complications in Vascular Surgery

### The combination of abdominal and vascular surgery in complex clinical-operative findings – possibilities and management of vascular reconstructive surgery in the course of abdominal surgical interventions

(Abstract ID: 354)

U. Barth^1^, Z. Halloul^1^, F. Meyer^1^, R. S. Croner^1^

^1^*Universitätsklinikum Magdeburg A.ö.R.*

**Background:**

The treatment of complex diseases in abdominal surgery (such as advanced, recurrent or multi-visceral tumor growth in pancreatic cancer, colorectal cancer, retroperitoneal sarcoma and others, respectively; acute mesenteric ischemia, intra-/postoperative bleeding) requires in the majority of cases the involvement of a vascular surgeon. The presentation of the vascular surgical reconstruction options should help to i) recognize problems and challenges early and ii) initiate appropriate treatment strategies.

**Materials and methods:**

Overview of own operative and clinical with vascular reconstructive possibilities in surgical approach and tactic as well as their management in complex visceral-surgical applications as well as their reflection in selected scientific references.

**Results:**

In advanced pancreatic carcinoma, reconstruction of veins is generally possible and feasible by various procedures (direct suture, short segmental resection, patch plasty, autologous venous bypass) according to standardized classification of (borderline) resectability (circumferential extension of tumor adherence/-infiltration) and adequate inclusion of the interdisciplinary tumor board decision on an optionally relevant neoadjuvant therapy. The arterial vascular reconstruction remains under ongoing discussion due to increased morbidity and low evidence level of reasonable prognostic benefit. In very selected individual cases (young age, urgent patient request, in the context of clinical studies), adequate response to neoadjuvant therapy, promising R0 resection status, reasonable risk-benefit ratio, proven and developed experience in complication management, certified "pancreas center", sufficient "hospital and surgeon's volume", established and experienced interdisciplinary surgical management and -operative tactic, etc.), it might be performed in (highly) specialized centers with associated proven vascular surgical expertise.- Even with extensive retroperitoneal sarcomas multivisceral resections, the reconstruction of the (possibly) infiltrated inferior vena cava (including endocaval tumor thrombi) or common iliac artery by a (autologous/xenogeneic) patch plasty or autologous bypasses (alternative: silver-coated alloplastic prosthesis material) are necessary.- Inflammatory processes, tumor adhesions / infiltrations of adjacent or neighboring/surrounding anatomical structures or vascular injuries after (e.g.) pancreatic or hepatobiliary surgery, complicated by anatomical variants, represent an extreme challenge for the surgeon.- For mesenteric ischemia, the prospect of successful operative treatment is substantially determined by the time factor. Immediate diagnosis-specific recanalization (by thrombectomy and/or desobliteration) and vascular reconstructive measures such as patch plasty, transposition or aorto-(iliaco-)mesenteric bypass with autologous removal of veins (alternative: silver-coated alloplastic prosthesis material) may have a decisive impact on the prognosis of the disease.

**Conclusion:**

Vascular reconstruction in abdominal surgery, especially tumor surgery, requires adequate vascular surgical expertise. In addition to high-standard preoperative diagnostics, developed specific vascular surgical / medical as well as interdisciplinary experience in case management and surgical tactics / technique, the knowledge of the possible reconstruction techniques and their optimal use are needed.

### Arterial fistulations associated with gastrointestinal (GJ) pathologies - a representative case series

(Abstract ID: 355)

U. Barth^1^, M. Pech^1^, F. Meyer^1^, Z. Halloul^1^

^1^*Universitätsklinikum Magdeburg A.ö.R.*

**Background:**

The treatment of specific diseases (such as arterial fistulas to gastrointestinal pathologies) is usually characterized by a non-evidence-based single-case treatment.

**Materials and methods:**

To provide case studies with casuistic presentations of the successful, interdisciplinary treatment of rare arterial fistulas to gastrointestinal pathologies.

**Results:**

1) A 70-year-old patient with AEG-typeII tumor lesion (extending intramurally within esophageal wall up to 25cm from teeth) underwent gastrectomy, splenectomy and esophageal resection with colonic-segment interposition (histology: pT2aN1[1/18]L1V1G3). Subsequent anastastomotic insufficiency was treated by implantation of a self-expanding stent but provoked hemorrhage into the oesophageal lumen from a high-flow aorto-esophageal fistula as suspected.

2) A 79-year-old patient with hematuria/bladder tamponade following former neoadjuvant radiochemotherapy of a suprasphincteric rectal cancer and rectum extirpation (ypT3ypN0M0) due to a uretero-iliac fistula. Treatment: Combination of vascular surgical/image-guided radiology with i) an AMPLATZER™ insertion into the right internal iliac artery and ii) right-common-iliac-artery stenting by cross-over maneuvers from the left-femoral-artery access side.

3) A 70-year-old patient received an aorto-aortic prosthesis for a mycotic aortic aneurysm; 7 years later, an "aneurysma spurium" of the distal anastomosis was approached by bi-iliac EVAR. After patient’s initial refusal of open surgery for recurrent infectious episodes, the prosthesis was finally explanted and a subclavio-bifemoral bypass was impanted. The primarily not intented bridging with EVAR provided a suitable interims solution to prevent an imminent aortointestinal fistula or rupture associated with severe blleding prior to definitive care.

4) A 72-year-old patient with unresectable T4-rectal cancer imposed with recurrent bleeding episodes out of the descendostoma (created palliatively). Angiography revealed bleeding out of the AIE (stenting) and AMI (coiling). Further, a sinking abscess of the right leg appeared most likely originating from perforated rectal cancer leading to: i) cancer-surrounding cavity was oppend and drained and ii) the suspected, Avastin-induced iliaco-(later: mesenterico-)enteric fistula was excised (including iliac stent removal & vascular ligation).

5) A 46-year-old male with peritoneal dialysis catheter infection due to acute necrotizing pancreatitis developed a "frozen abdomen". Upper gastrointestinal hemorrhage occurred (CT scan: bleeding into a pseudocyst) leading to minimally invasive CT-guided percutaneous thrombin instead of supraselective catheterization and coilng due simultaneous aortic dissection, which was a meaningful, feasible and safe alternative procedure for "bridging" (gain in time).

6) A 57-year-old patient with the diagnosis of a pseudoaneurysm of the left gastric artery including an arterio-venous fistula to the "vena coronaria ventriculi" underwent supra-selective catheterization and successful embolization with micro-coils (follow-up investigation: left gastric artery was still open and stenosis oft he coeliac trunc). Four pseudoaneurysms of the inferior pancreaticoduodenal artery were also successfully embolized.

**Conclusion:**

The appropriate management of arterial fistulas to gastrointestinal pathologies is extremely demanding & requires the interdisciplinary expertise of a tertiary vascular medical center combining various treatment modalities.

### Surgeon-modified fenestrated Stentgrafts for pararenal aneurysms - Video

(Abstract ID: 698)

N. Tsilimparis^1^, T. Kölbel^2^, F. Heidemann^2^

^1^*Uniklinik München*

^2^*Deutsches Aortenzentrum Hamburg*

**Background:**

As open surgical repair for complex aortic aneurysm is associated with a significant morbidity and mortality, fenestrated and/or branched stent grafts are the preferred surgical approach in high-risk patients. Due to a delayed delivery time up to 8 weeks, custom-made fenestrated/branched devices are not available in emergency setting.

Surgeon-modified endografts (sm-fbEVAR) could be an alternative for such patients.

**Materials and methods:**

We present a video of a modification of a thoracic endograft to create a 4-vessel fenestrated endograft on the back table.

**Results:**

The modification is made on the back-table during anesthesia induction of the patient. Duration of the modification is 90-120 min. The fenestrations are performed with thermic cautery and re-enforced with a radiopaque material. A constraining wire to reduce the diameter the stentgraft with constraining ties is performed. Resheathing is performed with use of Vicryl temporary suture. Tips and Tricks of the technique are discussed.

**Conclusion:**

sm-fbEVAR can be utilized for urgent symptomatic or contained-ruptured complex aortic aneurysms in high-risk patients with unsuitable anatomy for commercially available stent grafts.

**Picture:**
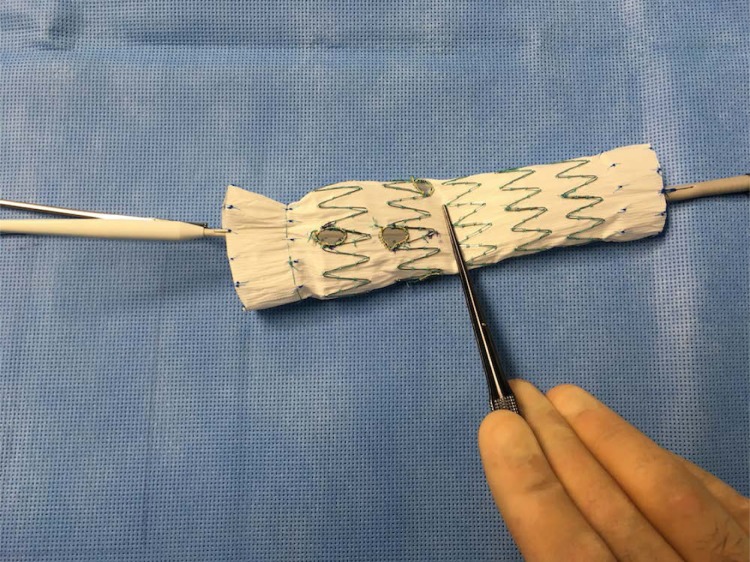


## DGG: A-Z: "Aorta, Screening, Centres"

### An Authentic Simulator as Alternative for Pre-Clinical Animal Models to Study New Approaches for Endovascular Interventions

(Abstract ID: 108)

M. Kaschwich^1^, F. Matysiak^1^, J. Bouchagiar^1^, A. Dell^1^, A.-C. Griesmann^1^, M. Horn^2^, J.-P. Goltz^1^, M. Kleemann^1^

^1^*Uniklinik Lübeck*

^2^*Prince of Wales Hospital, University of New South Wales, Randwick, Sydney*

**Background:**

Nowadays, endovascular procedures have become a standard repertoire of modern vascular surgeons and replace more and more conventional open surgery. Therefore the development of new endovascular approaches is of high clinical interest. To prevent humans form harm, animal models are part of the translational process of the establishment of new techniques into clinical practice. But on the other hand the directive of the European parliament and the council of the European Union says that "wherever possible, a scientifically satisfactory method or testing strategy, not entailing the use of live animals, shall be used instead of a procedure".

Purpose: The purpose of this project is the development of an authentic simulator for endovascular interventions that can minimize the use of animal models.

**Materials and methods:**

The goal of the project is the development of an authentic environment to simulate endovascular procedures as realistic as possible. Therefore, we build a simulator with exchangeable vascular pathologies. The 3D-vascular-models were printed via rapid prototyping using preoperative CTA-scans as a blueprint. The project was done in collaboration with the Fraunhofer Research Institution for Marine Biotechnology (EMB), who provided advanced 3D printing techniques, and HumanX, who supported us with constructing expertise.

**Results:**

We developed an authentic prototype with exchangeable vascular pathologies that can be used for endovascular research purposes as we do in our Nav EVAR project, were we try to implement a new navigation system for endovascular procedures. Furthermore it can be used for simulation and training of endovascular procedures.

**Conclusion:**

Authentic simulators for endovascular procedures can be used for the implementation of new endovascular techniques and research purposes to minimize the use of animal experiments, at least at an early stage of the study.

**Picture:**
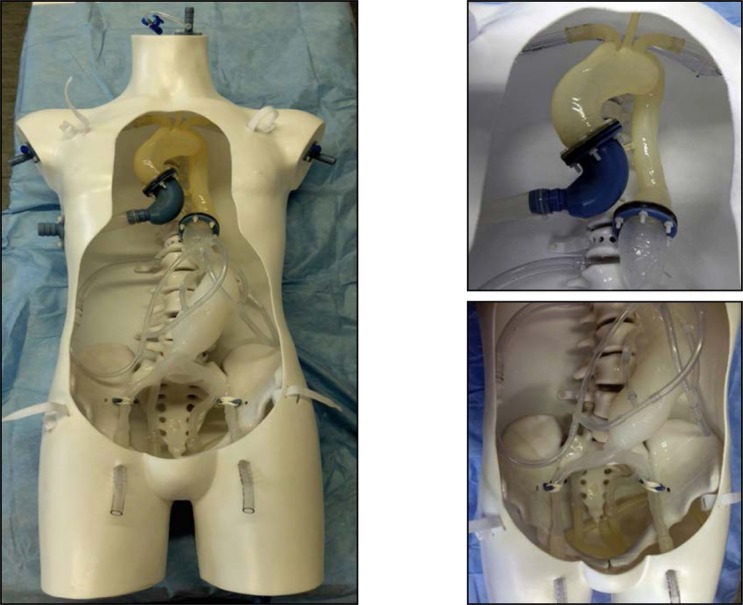
Prototype of Endovascular Simulator

### Inherited vasculopathies which patients benefit from genetic testing?

(Abstract ID: 394)

P. Erhart^1^, C. Grond-Ginsbach^1^, D. Böckler^1^

^1^*Universitätsklinikum Heidelberg*

**Background:**

Genetic variants may contribute to vascular diseases. The aim of this review is to present vascular pathologies with a suspected genetic background apart from the known hereditable connective tissue disorders.

**Materials and methods:**

Results from our department and cooperative centers involving acute aortic syndromes with type B dissection (n=105) and cervical artery dissection (CeAD, n=883) are presented. Known gene mutations affecting TGF-beta signaling (FBN1, SMAD3, TGFBR1, TGFBR2) and connective tissue integrity (COL3A1, COL5A1), age, vascular risk factors, familial history, comorbidities and clinical characteristics were examined and compared to control groups without genetic findings.

**Results:**

Apart from the known hereditary connective tissue diseases, genetic imbalance and mutations affecting the cardiovascular system are frequently seen in younger patients presenting with CeAD or type B aortic dissections. Certain single nucleotide polymorphisms (SNP) within the Low Density Lipoprotein Receptor-related Protein 1 (LRP1) and copy number variant (CNV) enrichment within specific cardiovascular genes were identified in type B aortic dissections and CeAD respectively. Incidence, inheritance and age at disease onset could be associated to specific genetic variants and mutations. However genetic disposition was not necessarily linked to the clinical course and disease progression in patients.

**Conclusion:**

It remains a challenging task to identify healthy individuals that benefit from genetic testing. Genetic consultation and testing however should be considered in young patients without cardiovascular risk factors suffering vascular diseases.

### Dare to C.A.R.E.: Implementation of a combined screening program for vascular diseases in Germany

(Abstract ID: 412)

U. Ronellenfitsch^1^, A. Peters^1^, M. Wortmann^1^, D. Böckler^1^

^1^*Universitätsklinikum Heidelberg*

**Background:**

The screening for vascular diseases with high prevalence might facilitate early treatment and thus lead to decreased disease-related morbidity and mortality. While ultrasound screening for abdominal aortic aneurysm (AAA) has been established for a high-risk group in Germany, there is no evidence regarding a combined screening for carotid stenosis, AAA and peripheral artery occlusive disease (PAOD). The "Dare to C.A.R.E." screening program comprises carotid ultrasound, abdominal aorta ultrasound and ankle-brachial index measurement.

**Materials and methods:**

The screening program has been implemented in the framework of a clinical study. The planned sample size of the study is 500 participants. Here, results regarding feasibility of the program implementation and utilization are presented.

**Results:**

The screening program is targeted at all persons above 60 years of age, at persons above 50 years of age with at least one established cardiovascular risk factor and at diabetics above 40 years of age. Potential participants are addressed at general practitioners or non-vascular specialist practices by means of information material which was provided to all practices in the hospital catchment area in a promotional roll-out. Moreover, press announcements were launched and information was provided on the hospital’s homepage in order to attract potential participants. The actual screening is offered during a once weekly clinic with a 30-minute time slot allotted to each participant. In the first four months after implementation, 122 persons have been screened with another 81 persons already scheduled for screening.

**Conclusion:**

A combined screening program for carotid stenosis, AAA and PAOD has been successfully implemented by inviting participants at general practitioner and non-vascular specialist practices and through local media. The uptake of the program was high. Conclusions regarding disease detection rate and utility of the screening as well as effects on hospital admissions related to screening results can be made once enrolment has been completed.

### Intramural Hematoma and Penetrating Aortic Ulcers – Natural History and Indications for Treatment

(Abstract ID: 473)

K. Meisenbacher^1^, D. Böckler^1^

^1^*Universitätsklinikum Heidelberg*

**Background:**

Aortic intramural hematoma (IMH) and penetrating aortic ulcer (PAU) represent entities within the disease-complex of acute aortic syndrome. Despite a relevant rate of progression to dissection and rupture, data addressing natural history are scant. While predictors of progression are known, guideline recommendations with respect to treatment are still based on low level of evidence. The aim of this review was to provide an overview of risk factors for progression and develop treatment strategies for uncomplicated/complicated courses of IMH/PAU with respect to current guidelines.

**Materials and methods:**

A selective literature research on the natural course and treatment indications in the light of current guidelines and expert recommendations on IMH/PAU was performed.

**Results:**

Selective literature research on natural course of IMH/PAU showed high mortality rates of up to 30-50%. Anatomic location of the lesion, maximum aortic diameter, persistent pain, hemodynamic instability, pleural effusion and IMH-thickness as well as secondary intramural lesions have been described as predictors for disease progression in IMH/PAU. A combination of these predictors, particularly, is mostly highlighted as a complicated course and may, in individual cases, lead to endovascular treatment.

**Conclusion:**

According to current guidelines, conservative management of IMH/PAU is indicated in uncomplicated cases. Predictors of progression are known and TEVAR can be applied in patients showing these predictors. Because long-term results for survival and treatment success are still missing, this remains an individual case selection.

### International Variations in Amputation Practice: A VASCUNET Report

(Abstract ID: 827)

C.-A. Behrendt^1^

^1^*University Medical Center Hamburg-Eppendorf (UKE)*

**Background:**

To study international differences in incidence and practice patterns as well as time trends in lower limb amputations related to peripheral arterial disease and/or diabetes mellitus.

**Materials and methods:**

Data on lower limb amputations during 2010-2014 were collected from population based administrative data from countries in Europe and Australasia participating in the VASCUNET collaboration. Amputation rates, time trends, in hospital or 30 day mortality and reimbursement systems were analysed.

**Results:**

Data from 12 countries covering 259 million inhabitants in 2014 were included. Individuals aged >= 65 years ranged from 12.9% (Slovakia) to 20.7% (Germany) and diabetes prevalence among amputees from 25.7% (Finland) to 74.3% (Slovakia). The mean incidence of major amputation varied between 7.2/100,000 (New Zealand) and 41.4/100,000 (Hungary), with an overall declining time trend with the exception of Slovakia, while minor amputations increased over time. The older age group (>=65 years) was up to 4.9 times more likely to be amputated compared with those younger than 65 years. Reported mortality rates were lowest in Finland (6.3%) and highest in Hungary (20.3%). Countries with a fee for service reimbursement system had a lower incidence of major amputation compared with countries with a population based reimbursement system (14.3/100,000 versus 18.4/100,000, respectively, p < .001).

**Conclusion:**

This international audit showed large geographical differences in major amputation rates, by a factor of almost six, and an overall declining time trend during the 4 year observation of this study. Diabetes prevalence, age distribution, and mortality rates were also found to vary between countries. Despite limitations attributable to registry data, these findings are important, and warrant further research on how to improve limb salvage in different demographic settings.

### Gender disparities in fenestrated and branched endovascular aortic repair

(Abstract ID: 828)

C.-A. Behrendt^1^

^1^*University Medical Center Hamburg-Eppendorf (UKE)*

**Background:**

Gender disparities in risk factors and outcomes following aortic repair are important issues in healthcare. To date, no large-scale multicentre study addresses this topic in complex endovascular aortic repair. We aimed to determine the outcomes following fenestrated or branched endovascular aortic repair of aneurysms and dissections in female and male patients.

**Materials and methods:**

Health insurance claims data of Germany’s third largest insurance provider, DAK-Gesundheit, were used to investigate gender disparities in elective fenestrated or branched endovascular aortic repair of thoraco-abdominal or abdominal aortic aneurysms or dissections performed between 2008 and 2017. Elixhauser comorbidities and the linear van Walraven score were used to adjust for confounders in the multivariable analyses.

**Results:**

There were 959 patients in the cohort, in whom 163 (17%) were female. The mean age was 73 ± 8 years with no differences between females and males. No gender disparities were observed regarding the van Walraven comorbidity score (6.9 vs 6.8, P = 0.83), but complications occurred more frequently in females. Acute renal failure (relative risk 1.71, 95% confidence interval 1.06-2.77), paraplegia (relative risk 2.71, 95% confidence interval 1.28-5.77) and bleeding or anaemia requiring transfusion (relative risk 1.76, 95% confidence interval 1.39-2.22) were more common in women. In multivariable models, female patients were at a higher risk of in-hospital death (odds ratio 3.206, P < 0.001). Consequently, female gender was associated with lower long-term survival (hazard ratio 1.506, P = 0.006).

**Conclusion:**

In complex endovascular aortic repair, females are more likely to experience complications and have worse in-hospital and, consequently, long-term survival when compared to males. Future studies should include anatomic parameters to determine the impact of anatomy on outcome disparities.

### Incidence, Predictors, and Outcomes of Colonic Ischaemia in Abdominal Aortic Aneurysm Repair

(Abstract ID: 829)

A. Larena-Avellaneda^1^, C.-A. Behrendt^1^

^1^*University Medical Center Hamburg-Eppendorf (UKE)*

**Background:**

Colonic ischaemia (CI) is a severe complication following abdominal aortic aneurysm (AAA) repair, leading to high morbidity and mortality. The aim of the study was to determine the incidence, predictors, and outcomes of CI following AAA repair.

**Materials and methods:**

National claims from Germany's third largest insurance provider, DAK-Gesundheit, were used to investigate CI after intact (iAAA) and ruptured (rAAA) AAA repairs. Patients undergoing endovascular (EVAR) or open surgical (OSR) repairs between January 2008 and December 2017 were included in the study.

**Results:**

There were 9145 patients (8248 iAAA and 897 rAAA) undergoing EVAR or OSR procedures and the median follow up was 2.28 years. Most patients were male (79.2% iAAA, 79.3% rAAA); the median age was 73.0 years (iAAA group) and 76.0 years (rAAA group). Overall, CI occurred 97 (1.2%) times after iAAA and 95 (10.6%) after rAAA. In univariable analyses CI occurred less often after EVAR than after OSR (0.6% vs. 3.7%; p < .001). Acute post-operative renal and respiratory insufficiencies were also related to the occurrence of CI (p < .001). CI was associated with greater in hospital mortality (42.2% vs. 2.7% for iAAA, 64.2% vs. 36.3% for rAAA; p < .001) and lower long-term survival for iAAA (Kaplan-Meier analysis). In multivariable analyses, rAAA (odds ratio [OR] 5.59), and higher van Walraven comorbidity score (OR 1.09) were independently associated with greater risk of CI occurrence. EVAR use (OR 0.30) was protective. EVAR use remained protective in stratified analyses within iAAA (OR 0.32) and rAAA (OR 0.26).

**Conclusion:**

Post-operative CI after AAA repair is not common but is associated with worse in hospital outcomes and lower long-term survival. EVAR was protective after both rAAA and iAAA repairs. When discussing the treatment of AAA with patients the protective effect of EVAR should be considered. Future studies should validate predictive scores and advance preventive strategies.

### ASA classification is a highly significant predictor of ipsilateral stroke free survival in asymptomatic octogenarians with elective internal carotid endarterectomy

(Abstract ID: 928)

P. Konstantiniuk^1^, G. Siegl^1^, K. Suppan^1^, G. Schramayer^1^, T. Cohnert^1^

^1^*Medical University of Graz*

**Background:**

Internal carotid endarterectomy in asymptomatic patients is being discussed controversially, in particular in octogenarians. The goal of this investigation was to determine, whether recently operated patients met the 2017 guidelines of the ESVS in hindsight.

**Materials and methods:**

Patients with operation between January 2011 and June 2013 were followed to determine their five year survival and stroke rate. Data was compared to all strong recommendations (Type I or III) from the ESVS guidelines. ‘Perioperative’ was defined as a 30 day interval following surgery. Stroke-free survival was determined with SPSS using the Kaplan Maier method. The influence of the following factors on stroke-free-survival was calculated with cox-regression: Gender, weight, height, BMI, hypertension, diabetes, PAD, coronary artery disease, cardiac rhythm, smoking habits, use of statins, renal function, ASA classification, age, operative technique, type of antithrombotic therapy, non-recent stroke.

**Results:**

Between 1.1.2011 and 30.6.2013 73 asymptomatic octogenarians underwent internal carotid endarterectomy at our University Hospital. There were 37 (50.7%) males and 36 (49.3%) females, mean age was 82.6 years (range 80.0 - 88.7 years), 71.2% (52) were under sufficient blood pressure lowering therapy, 15.1% (11) had insufficient treatment, 4.1% (3) had untreated hypertension and 9.6% (7) showed normal blood pressure, diabetes was present in 23.3% (17), previous cardiac infarction in 24.7% (18), previous CABG in 8.2% (6), non recent ipsilateral stroke in 8.2% (6) and non recent contralateral stroke in 17.8% (13).

There was one perioperative death (uneventful operation and hospital stay, 5 days after discharge the patient was admitted to another hospital for heart failure and died). Mean stroke free survival was 5.3 years (CI 4.7 - 5.8 years). During follow-up one patient experienced a minor ipsilateral stroke (Rankin 2). There were two factors with a significant impact on stroke free survival: ASA-classification (p = 0.004, ASA 1-3: 6.0 years, ASA 4: 4.7 years) and age (p = 0.01, 80-82: 5.5years, 83-89: 4.9years).

The current ESVS guidelines consist of 118 recommendations, 37 of those are applicable to the study population. The examined procedures, habits and results revealed, that 28 of 37 recommendations were met.

**Conclusion:**

Carotid endarterectomy in asymptomatic octogenarians is possible with a low perioperative stroke- and deathrate. Though our results are good, following the recommendations more closely might further improve results. Collecting more data and determining specific risk factors in this particular subgroup of patients will enable us to improve the selection process for internal carotid endarterectomy in asymptomatic octogenarians.

## DGG: Wounds / Vascular Surgery

### Stent infection as a possible cause of impaired wound healing in lower-limb amputation

(Abstract ID: 24)

A. Dadras^1^, C. Uhl^1^, T. Betz^1^, I. Töpel^1^, M. Steinbauer^1^

^1^*KH Barmherzige Brüder, Regensburg*

**Background:**

Stenting of the femoropopliteal segment has become a well-established procedure. Many patients who ultimately undergo a major amputation have received a stent angioplasty in the past. One of the recognized complications is stent infection, this, however, being very rare. We present two cases of infected nitinol stents in the superficial femoral artery after an above-knee amputation, causing major morbidity and multiple operative revisions.

**Materials and methods:**

Hospital-stay documents and data were collected from two cases. A systematic search of the Medline database was performed with the purpose of identifying other similar cases and results.

**Results:**

Surgical wound revision with removal of the stent was the treatment of choice, which stopped the local infection and led to secondary wound healing in both cases.

**Conclusion:**

The concomitant removal of stents could be considered in patients undergoing a major amputation. However, the role of its prophylactic removal to avoid secondary infections is unknown.

### Closed-incision negative-pressure therapy (cINPT) reduces surgical site infections in vascular surgery: A randomised control trial

(Abstract ID: 215)

A. Gombert^1^

^1^*Uniklinik RWTH Aachen*

**Background:**

Surgical site infections (SSI) of the groin remain a crucial problem in vascular surgery, prompting great interest in preventative techniques, such as closed-incision negative-pressure therapy (ciNPT). Benefits of ciNPT include reduced skin tension in the incision area, fluid removal, and the establishment of a barrier to external contamination during the first days after surgery. In this prospective randomised multicentre study, we aimed to assess the potential benefits of ciNPT application after groin incision for vascular surgery.

**Materials and methods:**

This study included 204 patients who underwent vascular surgery for peripheral artery disease (PAD) at two sites between July 2015 and May 2017. These patients received postoperative treatment with ciNPT (study group) or standard wound dressings (control group). After exclusion of drop-outs, 188 patients were assessed regarding wound complications and SSIs using the Szilagyi classification.

**Results:**

Mean patient age was 66·6 ± 9·4 years (range, 43-85 years), and 70 per cent were male (n = 132). Regarding PAD stage, 52 per cent were stage IIB, 28 per cent stage III, and 19 per cent stage IV. Among the patients, 45 per cent (n = 85) had undergone previous groin incision. The control group experienced more frequent SSI (33·3 per cent; 30/90) than the study group (13·2 per cent; 13/98; P = 0·0015; OR, 3·25; 95 per cent c.i., 1·5 to 7·4). The SSI incidence risk ratio was 0·4 in the study group compared to the control group.

**Conclusion:**

Our results confirmed a reduced SSI rate after vascular surgical groin incision using ciNPT compared to standard wound dressings.

### Alternative mobilization by means of a novel orthesis in patients with diabetic gangrene – first experiences with the "i-WALK 2.0^®^" (iWALKFree^®^ hands-free crutch for walking)

(Abstract ID: 358)

U. Barth^1^, K. Wasseroth^2^, Z. Halloul^1^, F. Meyer^1^

^1^*Universitätsklinikum Magdeburg A.ö.R., Magdeburg*

^2^*AMEOS Klinikum Schönebeck*

**Background:**

The mobilization of patients with diabetic foot syndrome after surgical treatment by debridement or partial amputation is usually difficult in daily practice and in inpatient care.

**Materials and methods:**

A Scientific case report - with a representative case showing the innovative possibilities of an alternative mobilization option by means of a new orthosis ("iWALK 2.0®") in PAOD after surgical therapy of concomitant diabetic gangrene, exemplified by the successful clinical course.

**Results:**

The inpatient admission of a 59-year-old male patient revealed septic gangrene of the right foot in insulin-dependent diabetes mellitus. After admission and initial diagnosis, the calculated antibiotics therapy and initial surgical rehabilitation of the right foot took place. After stabilization and control of the infection, the minor amputation was performed in the Bona-Jäger line without primary wound closure, the wound was closed by vacuum sealing. Complicated by pre-existing peroneal paralysis following herniated disc contralateral, mobilization could be accomplished out of the wheelchair using a novel "free-hand" orthesis "iWALK 2.0®" (IWALKFree®, Inc. Canada) and walker with physiotherapeutic support. Thus, while simultaneously relieving the operated foot, self-sufficient mobilization at a later time was possible. This gave the patient a positive attitude to life in addition to more independence.

**Conclusion:**

The successful application of the "free-hand" -site "iWalk 2.0®" under the listed clinical constellation thus suggests that it is a clear alternative of "postoperative rehabilitation" in the diagnosis of a surgically treated PAOD stage IV after minor amputation with a consolidated wound, ultimately one to achieve a more comprehensive level of experience for targeted use with a clearly defined indication will be achieved.

### Non-invasive perfusion monitoring with TIVITA hyperspectral camera system in peripheral artery disease

(Abstract ID: 435)

N. A. Sandkühler^1^, E. Grambow^1^, E. Klar^1^, M. Weinrich^1^

^1^*Universitätsmedizin Rostock*

**Background:**

Hyperspectral imaging (HSI) is a non-invasive, contact-free imaging technique to measure tissue oxygenation and bears great potential for screening and evaluation of therapy in peripheral artery disease (PAD).

**Materials and methods:**

In this study patients with PAD were assessed by means of the TIVITATM HSI camera system before and after surgical or endovascular revascularisation. For comparison with PAD patients an age-matched group (older than 50 years) not suffering from PAD was assessed. Furthermore, young study patients (18-35 years) were included to assess standard parameters.

HSI included assessment of tissue oxygenation (StO2) and subcutaneous near-infrared perfusion index (NIR) in the angiosome of the A. dorsalis pedis. Assessment of these parameters was performed using the camera-specific software package TIVITATM Suite that allows definition of different regions for analysis within the photographed tissue. In all patients HSI was performed prior to and one day after revascularistion.

Furthermore, ankle-brachial-index (ABI), complaint-free walking distance and skin temperature of the angiosome were assessed. Additionally, a questionnaire on the impact of PAD on psychological state and quality of life containing 25 questions with values between 1 (strongest reduction of the individual quality of life) and 7 (no reduction of the individual quality of life) was fulfilled by all study participiants.

**Results:**

ABI (1.13±0.07 and 1.24±0.12), skin temperature (35.64±0.22°C and 35.86±0.29°C) and vascular-quality-of-life score (169±0 and 160±13), in young and older participants respectively, did not show a statistical significant difference. The walking distance was not restricted in both groups. HSI measurements on the A. dorsalis pedis angiosome revealed StO2 of 39.5±6.77% and NIR of 39±9.3 in the young control group and StO2 of 38.5±6.67% and NIR of 40±8.2 in the older control group.

In patients with PAD ABI (0.71±0.43), vascular-quality-of-life score (88.41±29.46) and the complaint-free walking distance (70±51 m) but not skin temperature (36.02±0.42°C) were markedly decreased compared to the age-matched control group. Concerning this control group patients with respective endovascular and surgical revascularisations showed deficits in ABI (0.52±0.43 and 0.79±0.44), walking distance (91±29 m and 70±63 m) and vascular-quality-of-life score (91.3±29.2 and 81.6±34.0) before treatment.

In parallel, StO2 and NIR were reduced in all PAD patients (36.88±9.01% and 33.94±7.9, respectively). StO2 and NIR in the endovascular and surgically treated subgroups were comparable.

Postinterventionally, ABI (0.84±0.34), StO2 (41.1±9.84%) and NIR (37.2±8.97) increased in this subgroup. Surgically treated patients showed increased ABI (0.96±0.11), StO2 (42.4±5.68%) and NIR (37.2±7.69). Walking distance on post-procedural day was not assessable.

**Conclusion:**

The first results demonstrate that HSI can differentiate between patients with and without PAD. Furthermore, an increased tissue oxygenation after endovascular or surgical revascularization can be recognized. Cut off points for more precise prediction of the disease development are currently assessed in different angiosomes of the foot and lower limb and additional participants are recruited for the study. This might allow statistical quantification of these and other subgroups as well as establishing standard values for StO2 and NIR. Perspectively, HSI might be useful for screening of and follow-up in patients with PAD.

### Skin grafting over exposed bone in patients with peripheral arterial disease (PAD) stage IV

(Abstract ID: 753)

S. Khater^1^, T. Carus^1^

^1^*Asklepios Westklinikum Hamburg*

**Background:**

Wound management in patients with advanced PAD is a big challenge due to the poor blood supply of the extremities, large area affected with almost no granulation tissue and often persistent infections. The management ends frequently with amputation.

**Materials and methods:**

We present 3 cases of successful transplantation of split skin graft on large wounds with exposed bone or tendons in patients with stage 4 PAD with infected wounds, avoiding the need for further amputation.

In three cases we faced the challenge of dealing with infected large wound and exposed bone. The patients suffered from peripheral arterial diseases with poor perfusion and ABI values < 0.5.

The target was to preserve the limb in one patient and to avoid further amputation in the other two patients who previously underwent forefoot amputation. We started with antibiotic therapy, surgical debridement of the wound and negative-pressure wound therapy. To improve the blood supply percutaneous transluminal angioplasty (PTA) was performed with additional intravenous Alprostadil 40 micrograms twice daily.

**Results:**

In the following weeks the blood supply of the affected area improved leading to increasing granulation, covering the complete defect. Final split skin grafting ended the therapy with complete wound healing.In our three patients, complete wound closure could be achieved without need for amputations.

**Conclusion:**

In cases of PAD IV and infection of large wounds with exposed bone or tendons, a combined management of improving blood supply and vacuum therapy can establish granulation tissue to cover the whole defect. After split skin transplantation with complete wound healing amputations can be avoided in many cases.

